# Improving orthotopic mouse models of patient-derived breast cancer brain metastases by a modified intracarotid injection method 

**DOI:** 10.1038/s41598-018-36874-3

**Published:** 2019-01-24

**Authors:** Zongming Liu, Yanzhi Wang, Sheheryar Kabraji, Shaozhen Xie, Peichen Pan, Zhenning Liu, Jing Ni, Jean J. Zhao

**Affiliations:** 1 Department of Cancer Biology, Dana-Farber Cancer Institute, Boston, MA 02215 USA; 2 Department of Biological Chemistry and Molecular Pharmacology, Harvard Medical School, Boston, MA 02115 USA; 3 Key Laboratory of Bionic Engineering (Ministry of Education), Jilin University, Changchun, Jilin 130022 P. R. China; 4 Department of Anesthesiology, Jilin Cancer Hospital, Changchun, Jilin 130000 P. R. China; 5 Department of Medical Oncology, Dana-Farber Cancer Institute, Harvard Medical School, Boston, MA 02215 USA

## Abstract

Breast cancer brain metastasis (BCBM) remains a major clinical problem. Approximately 10–16% of patients with breast cancer develop brain metastases (BCBM). However, no systemic therapy has gained regulatory approval for the specific treatment of BCBM and this remains an area of persistent, unmet medical need. Rapid, predictive and clinically-relevant animal models are critical to study the biology of brain metastases and to identify effective therapeutic approaches for patients with BCBM. Here, we describe a method for efficient establishment of orthotopic mouse models of patient-derived brain metastases via an improved intracarotid injection protocol that permits tumor cell growth in the unique brain microenvironment without compromising the blood-brain barrier (BBB). We demonstrate that our newly improved models of patient-derived brain metastases recapitulate the histologic, molecular, and genetic characteristics of their matched patient tumor specimens and thus represent a potentially powerful tool for pre-clinical and translational research.

## Introduction

Very few cancer cells can spread to distant organs because they need to survive a series of highly selective events, termed the “metastatic cascade”^1^. Through this multi-step process, primary tumors cells acquire the ability to invade surrounding tissue, enter the bloodstream, extravasate from the bloodstream, pass through the blood-brain barrier (unique for brain metastasis), and colonize distant organs^2–9^. Therefore, metastatic colonies most likely originate from those cells (amongst the highly heterogeneous primary tumor cell population), that have acquired the ability to overcome each step of the metastatic cascade and survive at the distant metastatic sites^1,7,10–12^.

Given the myriad adaptations that tumor cells undergo to reach and grow at a metastatic site, it is therefore not surprising that findings from pre-clinical animal studies often fail to recapitulate the complexity of tumor biology in patients, and drug responses in such models often cannot be validated in human clinical trials^13^. To improve the clinical relevance of animal models, such models must faithfully represent the microenvironment and the cellular diversity of patient tumors. To that end, we propose that orthotopic injection of patient-derived BCBM cells into the murine brain may better replicate patient biology, compared to classical xenograft-based approaches involving subcutaneous transplantation of i*n vitro* cultured cell lines^14^.

A number of approaches for generating murine-based breast cancer brain metastasis models currently exist. However, these models have a number of key technical limitations that hinders their usefulness for application towards preclinical studies. For example, in cell line xenograft models that do metastasize, resulting satellite tumors tend to form at extracranial sites, such as the lung or bone, as opposed to the brain. While injection of cancer cells into the tail vein (intravenous) or to the heart (intracardiac) does somewhat increase the frequency of tumor formation in the brain, the overall rate of these events remains low, and mice typically die of metastases to other sites^5,7,15,16^. Injection of cancer cells directly into the brain (stereotactic orthotopic injection) or to the internal carotid artery which supply the brain (intracarotid) have very high success rate of forming brain metastases and are thus suitable for use to interrogate cancer biology and for preclinical drug screens^17–20^. Stereotactic orthotopic injection is the most popular and reliable approach partly because the difficulty of the injection technique is moderate^17,18^. Intracarotid injection requires the tumor cells to penetrate the brain-blood barrier before entering the brain, therefore more physiological relevance than stereotactic orthotopic injection^20^. However, intracarotid injection is extremely technically challenging, and still shows some degree of tumor cell deposition at unintended sites. For example, the success rate of producing intracranial melanoma metastases by intracarotid injection differs depending on cell line and whether the internal carotid artery (ICA) or external carotid artery (ECA) is used^21^. Here, we describe a significantly improved protocol for intracarotid injection for generating orthotopic PDX models of BCBM that overcomes these latest challenges.

## Results

### Improved intracarotid injection protocol

**Figure 1:**
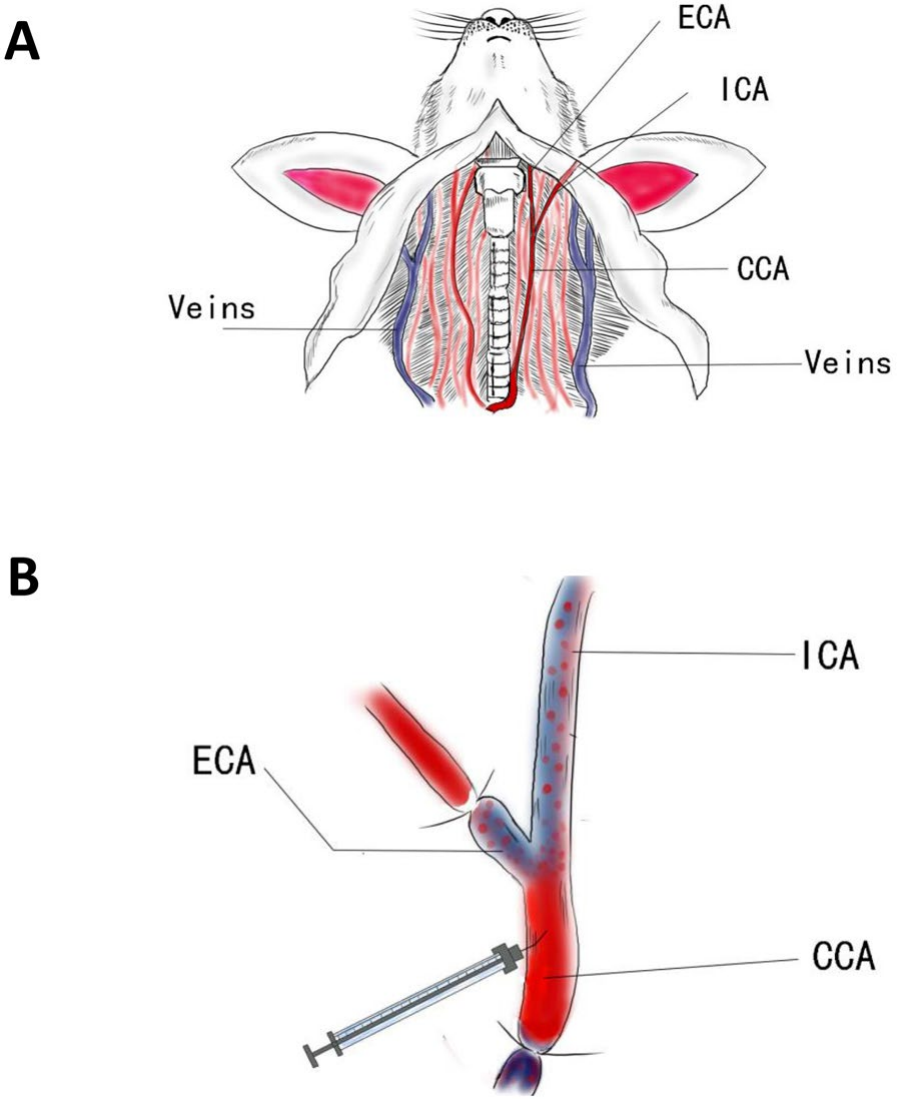
(**A**) Schematic depicting the mouse neck with vascular structure. ICA, internal carotid artery; ECA, external carotid artery; CCA, common carotid artery. (**B**) Schematic drawing of carotid ligations and injection. The branches of ECA and bottom of CCA were ligated while leaving ICA open for injecting cells.

General protocols of intracarotid injection of tumor cells to establish experimental models of brain metastases have previously been reported^21–23^. In these approaches, dish-cultured cancer cells are typically injected into the mouse internal carotid artery, where the cells then metastasize into the brain. However, in our experience, tumor cells may also transit through the branches of the external carotid artery forming metastatic deposits in the face, the ears, or the facial skin. Indeed, we found that several mice had obvious ear and/or face inflammation hours after receiving intracarotid injection of primary BCBM PDX tumor cells (data not shown). It is likely that tumor cells migrate to those areas causing inflammation that resulted in mice reaching prespecified humane end point. Eventually, we failed to establish PDX models for DF-BM#656, a BCBM sample by this conventional intracarotid injection method. Because previous reports do not describe tumor formation along branches of the external carotid artery, one possibility is that BCBM PDX tumor cells may have a higher predisposition to form metastases at extracranial sites than dish-cultured cell lines^23^. To solve the problem of unintended tumor cell deposition along the branches of the external carotid artery, we developed an improved protocol based on the previous established one^23^ with higher success rate and less toxicity. Specifically, we found that anterograde ligation of the external carotid artery with retrograde ligation of the common carotid artery (relative to the injection site) results in improved tumor cell migration towards, and seeding within, the brain (Fig. 1). Ligation of the common carotid artery prevents cancer cell migration towards the heart, reducing tumor formation at more distant organs, while simultaneous ligation of the external carotid artery directs cancer cells intracranially to improve the frequency of tumor seeding in the brain and reduce inflammation/tumor formation toxicity along the distribution of the ECA e.g. the facial artery. In this improved protocol, ligation of the external carotid artery is the most difficult step, as particular care needs to be exercised to avoid damaging the vagus nerve or bulbus caroticus, which likely result in sudden and fatal cardiac and respiratory arrest.

### Establishing BCBM PDXs by improved intracarotid injection protocol

**Figure 2:**
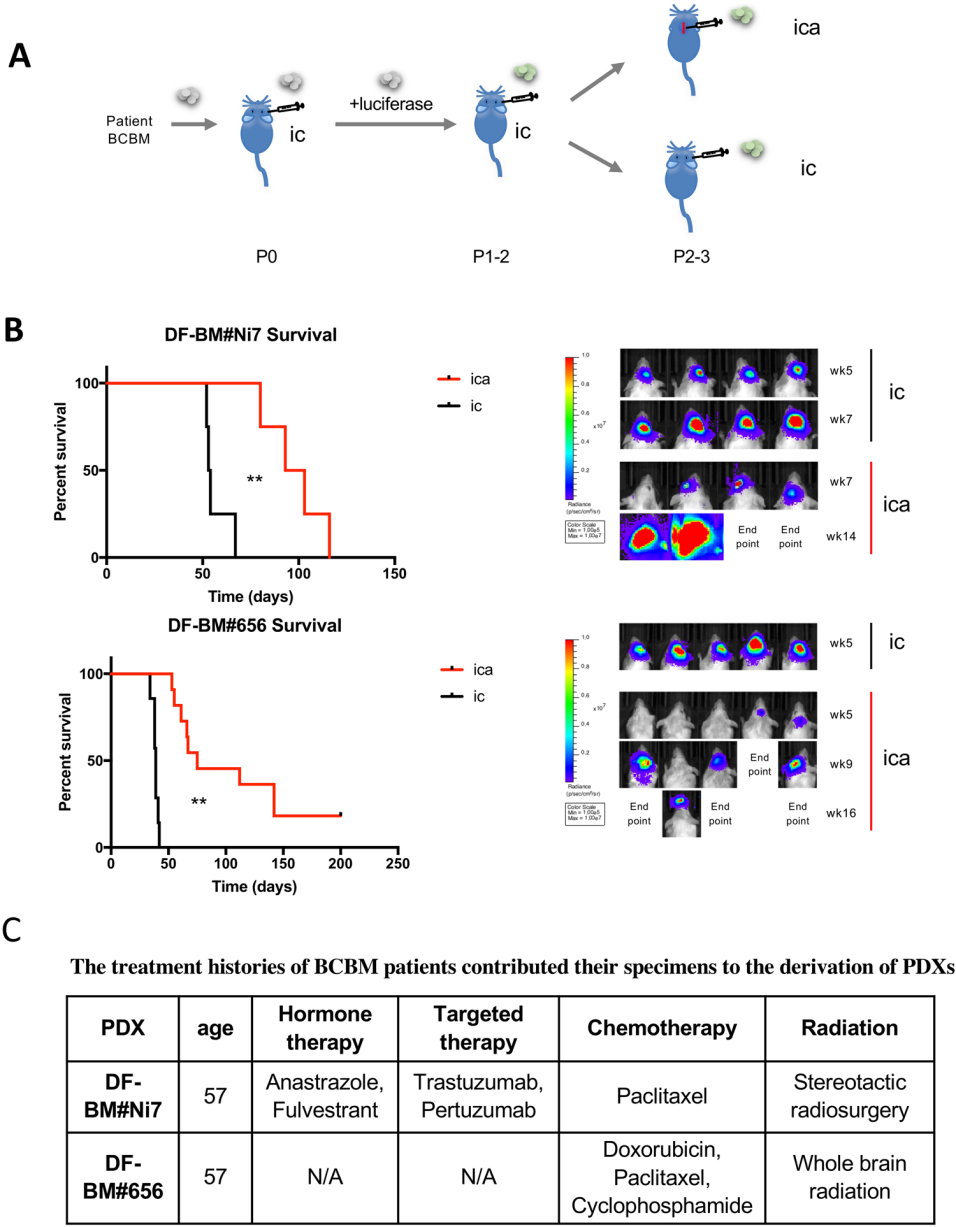
(**A**) Schematic depicting the process of generating BCBM PDXs in this study. (**B**) Left panel: Kaplan–Meier survival curves of DF-BM#Ni7 and DF-BM#656-bearing mice by intracarotid artery injection (ica, red) compared to intracranial injection (ic, black). **P < 0.01. Right panel: Bioluminescence imaging analysis of mice bearing DF-BM#Ni7 and DF-BM#656 tumors at indicated time after injecting. (**C**) The treatment histories of BCBM patients who contributed their specimen to the derivation of PDXs.

With this improved method of intracarotid injection via the internal carotid artery, we were able to efficiently establish PDX tumors that are suitable for preclinical biological research. To monitor tumor growth in the brain, the primagraft at passage P0 were freshly dissociated, transduced with luciferase gene, and re-injected intracranially into P1 mice to propagate. The following established primagrafts expressing luciferase were then re-injected into additional cohorts by intracranial injection (ic) or intracarotid artery injection (ica) (Fig. 2A). Not surprisingly, tumor-bearing mice by intracarotid artery injection tend to have delayed bioluminescence signaling and survive longer. The median survival of mice bearing DF-BM#Ni7 are 54d and 98d by ic and ica, respectively; while those for DF-BM#656 are 39d and 75d by ic and ica, respectively (Fig. 2B). The longer survival time of mice with ica is likely due to significant tumor cell attrition in the process of migrating to and colonizing the brain which also takes longer time than direct intracranial injection^1^. The take rates of DF-BM#Ni7 and DF-BM#656 were 100% (4/4) and 82% (9/11), respectively, via this improved intracarotid artery injection method (Fig. 2B).

### BCBM PDXs phenocopy parental patient tissues

**Figure 3:**
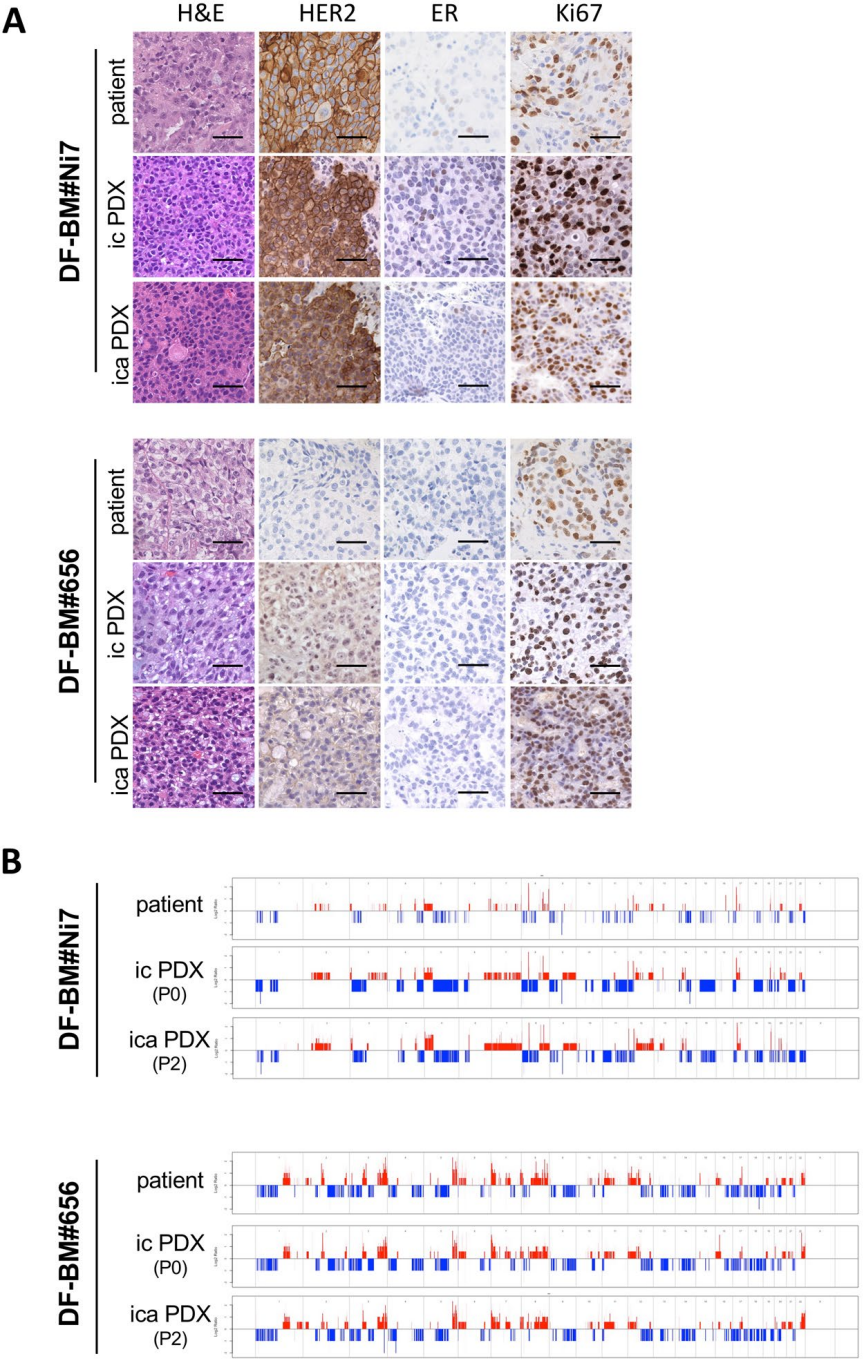
(**A**) Histologic and immunophenotypic analysis of HER2, ER, and Ki67 on the orthotopic brain metastatic PDXs (top panel: DF-BM#Ni7; bottom panel: DF-BM#656) by intracranial injection (ic PDX) or by intracarotid artery injection (ica PDX), and their matched patient specimens (Scale bars = 50 μm). (**B**) Patterns of genome-wide DNA copy number variations in orthotopic brain metastatic PDXs (top panel: DF-BM#Ni7; bottom panel: DF-BM#656) by intracranial injection (ic PDX) or by intracarotid artery injection (ica PDX), and their matched patient specimens.

Similar to PDX models established using intracranial injection, PDX tumors established by intracarotid artery injection resemble to the parent breast metastatic brain tumors. DF-BM#Ni7 PDXs (via both ica and ic) retained expression of HER2 and ~1% ER-positive cells, comparable to the matched patient tumors. Similarly, DF-BM#656 PDXs and matched parent tumor were negative for HER2 and ER (Fig. 3A). Whole exome sequencing results revealed both ica PDXs and ic PDXs largely maintained genome-wide DNA copy number variations compared to the parental patient tumors (Fig. 3B). Taken together, these data suggested that DF-BM#Ni7 and DF-BM#656 BCBM models accurately recapitulate the histological, molecular, and genomic levels of the original patients’ tumors.

## Discussion

Cancer cell clones that establish metastases have evolved to survive the many steps of the metastatic cascade. Importantly, the genomic and phenotypic adaptations of brain metastases are likely to be distinct from those of the primary tumor and could be clinically actionable^18,24^. Establishing BCBM PDX animal models through either intracranial injection or intracarotid injection will allow interrogation of many targets and may be capable of predicting the clinical activity of these novel agents in cancer patients. Choosing between either approach will depend on the scientific question under examination, e.g. drug sensitivity *in situ* vs interrogating mechanisms of metastasis. In this report, we provide a modified and improved intracarotid injection protocol that allowed us to establish orthotopic PDX mouse models from primary breast cancer brain metastasis as reliable platforms for mechanical dissection and drug screening. The use of such techniques will increase our knowledge of the metastatic process and help identify new targets of cancer metastasis.

## Materials and Methods

### Patient-derived xenograft

The informed consent was obtained from breast cancer patients, brain tumor samples were acquired from breast cancer patients undergoing neurosurgery at the Brigham and Women’s Hospital, and all research and methods were performed in accordance with relevant guidelines/regulations, as part of an Institutional Review Board (IRB) approved protocol (DFCI IRB 93-085 and 10-417). All the animal experiments were done in accordance with NIH animal use guidelines and protocols approved by the Dana-Farber Cancer Institute Animal Care and Use Committee.

The freshly resected human brain tumors were dissociated with Collagenase/Hyaluronidase (Stemcell Technologies), and the tumor cells were transplanted into the mice by intracranial injection immediately or after short-time *in vitro* culture within 5 days. The tumor cells were cultured in NeuroCult NS-A media (Stemcell Technologies) supplemented with heparin sulfate (2 mg/mL), Epidermal Growth Factor (EGF, 20 ng/ml), basic Fibroblast Growth Factor (bFGF, 20 ng/ml), and Hydrocortisone (0.5 μg/ml).

Tumors collected from mice bearing orthotopic PDXs were dissociated by mechanical pipetting and then cryopreserved. To propagated PDX lines, the frozen tumor cells were thawed and cultured overnight. Immediately before the injection, the tumor cells were further dissociated by Accutase (Sigma) and 100,000 cells were suspended in 1–2 μl PBS for intracranial injection or in 50 μl PBS for intracarotid artery injection.

#### Intracranial injection

Patient-derived xenografts were established by intracranial injection as described previously^18^. In brief, SCID female mice (~8 weeks old) (Taconic) (IcrTac:ICR-Prkdcscid) were anesthetized with ketamine/xylazine and placed in the stereotactic frame using ear bars. 100,000 cells (fresh human brain metastatic breast cancer cells or PDX cells) suspended in PBS (1–2 μl) were injected intracranially in the right striatum (2 mm to the right and 2.5 mm to the depth of the bregma).

#### Intracarotid injection

Mice were anesthetized using 100 mg/kg ketamine and 10 mg/kg xylazine, restrained on their backs, and placed under a dissecting microscope. Fur was shaved from the neck area after which underlying skin was disinfected with antiseptic iodine solution. Next, a midline, vertical, 1-cm skin incision was made, and the subcutaneous tissue, muscles and fat underwent blunt dissection using forceps to expose the left carotid sheath under the microscope. The left common carotid artery (CCA) was carefully separated from the surrounding nerves. Using 5.0 silk, a ligature was placed caudal to the injection site on the CCA, to block blood flow. The left external carotid artery (ECA) and internal carotid artery (ICA) were then separated from the carotid sinus and a second ligature was placed on the portion of the ECA rostral to the CCA injection site. A third ligature was loosely placed rostral to the injection site on the CCA. Next, a small piece of sterile PBS wet cotton was placed beneath the common carotid artery injection site, to immobilize the artery. A cancer cell suspension (10^5^ cells in 50 μl PBS) was injected slowly (over 2 min) into the CCA at a point just distal to the caudal ligature using a 33 Gauge needle (Roboz IN-942, bent 45 degree) (Fig. 1B). Upon completing the injection, the loose ligature rostral to the injection site was tightened prior to the withdrawing the needle. Finally, excess sutures were trimmed, muscles and fat were replaced, the skin was closed, and mice were allowed to recover in a warm and dry cage. The observed mortality rate due to complications during the procedure is less than 5% with proficient surgical skills.

### Lentiviral transduction to introduce Luciferase gene

Lentivirus were produced as described previously^18^. Fresh PDX tumors were isolated and dissociated, then transduced with lentiviral luciferase (HIV-Luc-ZsGreen, addgene#39196) in suspension with polybrene 8 μg/ml, in NeuroCult NS-A media (Stemcell Technologies). Three days later, the tumor cells were then propagated in mice.

### Bioluminescence imaging

In live mice, bioluminescence signals from luciferase after intraperitoneal injection of D-luciferin (80 mg/kg) (Gold Biotechnology) were recorded with IVIS Lumina III Imaging System (PerkinElmer). The signals were analyzed with Living Image Software (PerkinElmer).

### Immunohistochemistry

Immunohistochemical staining was performed as described previously^18^. Anti-HER2 antibody (OP15) was from EMD Millipore, anti-ER antibody (RM9101) was from ThermoFisher, and anti-Ki67 antibody was from DAKO (Carpinteria, CA).

### Whole exome sequencing

The whole exome sequencing (Ion Proton platform, ThermoFisher) and data analysis for CNV alterations were described previously^18^.

### Statistical analysis

The log-rank (Mantel-Cox) test (Prism) was used for statistical analysis of animal survival. *P < 0.05. **P < 0.01.

## Data Availability

All data are available from the corresponding author on reasonable request.
